# Editorial: *Pseudomonas* and *Acinetobacter*: From Drug Resistance to Pathogenesis

**DOI:** 10.3389/fcimb.2018.00068

**Published:** 2018-03-13

**Authors:** Ghassan M. Matar

**Affiliations:** Department of Experimental Pathology, Immunology and Microbiology, Center for Infectious Diseases Research, Faculty of Medicine, American University of Beirut, Beirut, Lebanon

**Keywords:** *Pseudomonas*, *Acinetobacter*, pathogenesis, antimicrobial resistance, drug resistance, microbial

The serious implications of *Pseudomonas* and *Acinetobacter* in healthcare setting outbreaks, along with their antimicrobial-resistant propensities, make it clear that these genera pose an imminent threat to public health. Both *Pseudomonas* and *Acinetobacter* employ numerous virulence factors that enhance their success as pathogens: biofilm formation, surface lipopolysaccharide, outer membrane proteins, secretion systems, etc. With respect to resistance, both microorganisms are intrinsically resistant to several classes of antibiotics. Furthermore, they can additionally acquire mobile genetic elements that confer further antimicrobial resistance; thus, pan-drug resistant isolates can be identified. Therefore, better understandings of the epidemiology, virulence, pathogenesis, and resistance patterns of and possible therapeutic options for these microorganisms are in constant demand.

When surveying *Acinetobacter*, Dahdouh et al. characterized 90 clinical isolates of *Acinetobacter baumannii* for their clonality, growth rates, antimicrobial susceptibility profiles, virulence factors, and mechanisms of carbapenem resistance. They discovered that most of their isolates belonged to international clone (IC) II, and had a high rate of carbapenem resistance; *bla*_OXA−23−like_ was the most common resistance gene. Despite these common features, there was no specific pattern of virulence, antimicrobial susceptibility profile, or growth rate associated with a clone. However, an association was found between surface motility, biofilm formation, and siderophore production. Taken together, these results imply that each *Acinetobacter* infection should be dealt with as a unique case, despite the presence of possible common clonality or antimicrobial resistance patterns. Additionally, a retrospective observational study by Ballouz et al. analyzed clinical outcomes and risk factors to predict mortality of patients with *Acinetobacter* bacteremia. It was observed that 91.1% of *A. baumannii* isolates causing bacteremia were extensively drug resistant (XDR). 75.3% of patients with *A. baumannii* bacteremia showed an Eastern Cooperative Oncology Group (ECOG) performance status of 4. Moreover, 70.37% of enrolled patient deaths were directly related to that bacteremia. No statistically significant differences in demographic, XDR profile, or source of bacteremia between survivors and non-survivors were identified; however, risk factors independently associated with mortality due to *A. baumannii* bacteremia included high-dose steroids and septic shock

In terms of virulence factors, the review by Moradali et al. highlighted the clinical importance of *Pseudomonas aeruginosa* infections in cystic fibrosis (CF) patients and as a causative agent of hospital-acquired infections. Furthermore, the review described the most recent findings on adaption mechanisms employed by *P. aeruginosa* for survival; discussed topics included the quorum sensing (QS) system, biofilm formation, antimicrobial resistance mechanisms, adaptive radiation, stringent responses, and resistance to foreign DNA. In addition, a review by Hotterbeekx et al. discussed the interaction between *P. aeruginosa* and *Staphylococcus* spp. *in vitro* and *in vivo*. The presence of Gram-positive bacteria increases the production of extracellular virulence molecules by *P. aeruginosa*, which presumably serves as a defense mechanism. However, these molecules also damage host tissue, especially in the case of CF patients. On the other hand, *Staphylococcus aureus* adapts to *P. aeruginosa* by switching to the small colony variant (SCV) phenotype. This switch results in slowed cellular growth and cellular functions that result in increased survival in unfavorable conditions. In a multi-species biofilm, *S. aureus* can resist antibiotics by benefitting from the *P. aeruginosa* biofilm. As a result, the co-presence of *Staphylococcus* spp. and *P. aeruginosa* can worsen disease outcome.

On the other hand, the review by Lee et al. extensively characterized multiple biological aspects of *A. baumannii*, including virulence factors, antimicrobial resistance mechanisms, and possible treatment options. Pathogenic characteristics covered included porins, capsular and lipopolysaccharides, phospholipases, outer membrane vesicles, metal acquisition, biofilm formation, and protein secretion systems. Antimicrobial resistance mechanisms included efflux pump upregulation, β-lactamases of several classes, membrane permeability defects, and aminoglycoside-modifying enzymes. Possible treatment options encompassed carbapenems with β-lactamase inhibitors, tetracyclines, polymyxins, and phage therapy. Furthermore, Rumbo-Feal et al. analyzed the products of the gene cluster A1S_0112-A1S_0119 of *A. baumannii* ATCC 17978. They were able to verify that A1S_0112-A1S_0119 represents a polycistronic operon consisting of eight genes. Additionally, they determined that the A1S_0114 gene is associated with acetin 505 (Ac-505), which is needed for cell adherence. Three animal model studies showed that *A. baumannii* with an A1S_0114 gene deletion experienced significantly reduced virulence and cell adherence; thus, it was concluded that the A1S_0114 gene is involved in *A. baumannii* pathobiology.

When studying pathogenic behavior, López et al. investigated the effect of bile salts on the growth, virulence, and gene expression of *A. baumannii* clinical isolates and isogenic mutant strains that lack the AdeABC efflux pump. They reported that bile salts activated the *A. baumannii* QS system and modulated surface motility, biofilm formation, and the bacterial Type VI secretion system. In addition, bile salts also increased *A. baumanniii* invasiveness. As for *P. aeruginosa*, Bielen et al. made use of the chronic pneumonia agar bead model to examine innate immunity in relation to biofilm-like structures. They demonstrated that such a model in rats can activate the same anti-inflammatory type 2 (Th2) cytokines and innate immune cells that are also documented in CF patients; thus, these authors concluded that these cytokines can be used as biomarkers for infections in CF patients.

When studying resistance patterns, Hua et al. employed a multi-omics approach to examine colistin resistance mechanisms. Specifically, colistin resistance was induced in an *A. baumannii* clinical isolate and then whole genome sequencing (WGS), transcriptome and real-time quantitative PCR analysis, proteomics analysis, and growth rate studies were performed on the isolate. It was found that the growth rate of the mutant isolate was slower than that of the original strain. In addition, WGS showed the presence of ISAba1 upstream of *lpxC* in the mutant strain but not in the original isolate. Transcriptome and real-time quantitative PCR analysis revealed that 137 genes showed significant differential expression following induced resistance. Finally, proteomic analysis showed that while the expression of the AdeABC efflux pump was upregulated, certain biochemical pathways were downregulated in the mutant strain as compared to the original isolate.

Lu et al. explored the implications on conjugation of the interaction between the QS system of *P. aeruginosa* and the regulatory protein SdiA of *Escherichia coli*. They concluded that QS-SdiA interplay exerts an inhibitory effect on conjugation; the loss of *lasI* and *rhlI* genes in *P. aeruginosa* and the *sdiA* gene in *E. coli*, promoted the conjugation process. In addition, the authors concluded that repressed relaxase gene expression in donor cells might be the mechanism behind AHL inhibition of conjugation.

When identifying therapeutic options, Soudeiha et al. employed the checkerboard, time-kill curve, and perpendicular E-test assays to assess the effect of colistin and carbapenem combinations against MDR *A. baumannii* isolates that harbor several OXA-type carbapenemases. They found that while the checkerboard and perpendicular E-test assays show an additive effect for colistin and carbapenems, the time-kill curve assay showed a significant bactericidal effect for colistin and imipenem as compared to colistin and meropenem. In a different light, Du et al. demonstrated the bactericidal activity of the human salivary protein Histatin 5 (Hst 5) against ESKAPE pathogens, especially *P. aeruginosa* and *A. baumannii*. They concluded that Hst 5 exerts its bactericidal effect on *Enterococcus faecium* and *Enterobacter cloacae* upon internalization into the cell and on *P. aeruginosa* and *A. baumannii* through membrane disruption. The effect was delayed, but non-lytic, against *S. aureus*. Furthermore, Hst 5 was ineffective in killing *Klebsiella pneumoniae*. Their findings suggest the possibility of using Hst 5 as prophylactic topical antimicrobial therapy to prevent surgical, burn, and wound infections.

*En masse*, the articles included in this research topic help to shed light on the growing problems of drug resistance and pathogenesis of *Pseudomonas* and *Acinetobacter*.

## Author contributions

The author confirms being the sole contributor of this work and approved it for publication.

### Conflict of interest statement

The author declares that the research was conducted in the absence of any commercial or financial relationships that could be construed as a potential conflict of interest.

